# Characteristics of Suicidal Patients Who Engaged in Suicide-Related Internet Use in the United Kingdom: Cross-Sectional Survey Findings

**DOI:** 10.2196/73702

**Published:** 2025-09-05

**Authors:** Lana Bojanić, Isabelle M Hunt, Saied Ibrahim, Pauline Turnbull, Sandra Flynn

**Affiliations:** 1 University of Manchester, 2nd Floor Jean McFarlane Building, Oxford Road, Manchester, England, M13 9PL, United Kingdom, +44 01612750709

**Keywords:** suicide-related internet use, suicidality, mental health patients, online behaviour, suicide prevention, mental illness

## Abstract

**Background:**

Suicide-related internet use encompasses various web-based behaviors, including searching for suicide methods, sharing suicidal thoughts, and seeking help. Research suggests that suicide-related internet use is prevalent among people experiencing suicidality, but its characteristics among mental health patients remain underexplored.

**Objective:**

This study aimed to examine the sociodemographic, clinical, and suicidality-related characteristics of suicidal mental health patients who engage in suicide-related internet use compared with those who do not.

**Methods:**

A cross-sectional survey was conducted from June to December 2023, recruiting participants aged 18 years and older with recent contact with secondary mental health services in the United Kingdom. The survey assessed sociodemographic characteristics, psychiatric diagnoses, suicidal thoughts and behaviors, and engagement in suicide-related internet use. Statistical analyses included chi-square tests, Wilcoxon tests, and multivariable logistic regression to identify predictors of engaging in suicide-related internet use.

**Results:**

Of 696 participants, 75% (522) engaged in suicide-related internet use in the past 12 months. Those who engaged in suicide-related internet use were almost 3 times as likely to have attempted suicide in the past year (32.5% vs 9.2%, *P*<.001). They were more likely to have a diagnosis of personality disorder (34.4% vs 18.5%, *P*<.001) and to disclose suicidal thoughts to someone (87.8% vs 72.8%, *P*<.001). They also reported higher levels of suicidal ideation intensity (median =6.6 vs 5.1, *P*<.001). There were no significant sociodemographic differences between groups, including age.

**Conclusions:**

The findings suggest that suicide-related internet use is a common behavior among suicidal mental health patients across various age groups, challenging the notion that it is primarily a concern for younger populations. The association between suicide-related internet use and increased suicidality highlights the need for clinicians to incorporate discussions about web-based behaviors in suicide risk assessments. Given the high rate of disclosure of suicidal thoughts among suicide-related internet users, clinicians may have an opportunity to engage in open, nonjudgmental discussions about their patients’ internet use.

## Introduction

For people experiencing suicidal thoughts and behaviors, suicide-related internet use can encompass a wide range of behaviors, including web-based searching for information about suicide methods, sharing suicidal thoughts, and seeking help [1]. Suicide-related internet use has become an important focus of research due to its potential influence on suicidal ideation and behavior. Understanding how people who engage in suicide-related internet use differ from those who do not is important for designing interventions, such as targeted web-based resources to promote help-seeking, improving digital literacy programs, and enhancing clinical strategies for suicide prevention.

Due to the inherently private nature of suicide-related internet use, precise prevalence rates of engagement in this behavior are not available; instead, estimates rely on cross-sectional and longitudinal studies. These studies reveal suicide-related internet use to be prevalent among suicidal people, with proportions of people engaging in it varying between 36% and 59%, depending on the population and timeframe studied, but often characterized by small sample sizes and focus on young people, often university students [2-5]. Therefore, the current literature does not fully represent the whole population of those who engage in suicide-related internet use. Previous research has consistently found differences between those who did and did not engage in suicide-related internet use, most notably that those engaging in suicide-related internet use tend to be younger and have higher levels of suicidal ideation [2-5]. This has also been reported among those who engage in suicide-related internet use only to seek help [6].

Mental health patients, in particular, represent a vulnerable and underresearched group when it comes to using the internet for suicide-related purposes. This group has closer access to treatment and support due to their engagement with health care services, and they experience significantly higher rates of suicide compared with the general population [7]. Therefore, their engagement in suicide-related internet use warrants closer investigation to uncover unique factors associated with this behavior and intervention opportunities. A 2018 UK study identified contrasting patterns of internet use between young people in the community and young self-harm patients [8]. Young community people ambivalent about suicide exhibited shifting between help-seeking and accidental exposure to web-based prosuicide content, often seeking connection. In contrast, self-harm patients with stronger suicidal intent engaged in calculated, methodical searches for lethal means, avoiding support resources entirely. This behavior aligns with the Interpersonal Psychological Theory of Suicide, particularly the concept of acquired capability, suggesting that specific web-based behaviors, such as focused search or avoidance of help sites, may signal elevated risk and should be evaluated by clinicians [9].

Proportions of mental health patients who have engaged in suicide-related internet use vary from 8% in patients who died by suicide to 27% of adult and 43% of adolescent psychiatric in-patients [10-12]. A case-control study on suicide-related internet use in mental health patients who died by suicide found those who engaged in this behavior were more likely to be younger (aged 25 years or younger), have a diagnosis of autism spectrum disorder, and have a history of childhood abuse [10]. In samples of both adult and adolescent hospitalized patients with affective disorder, those who engaged in suicide-related internet use were more likely to be younger and to have a higher incidence of suicide ideation and suicide attempts [11,12]. Patients who were hospitalized for self-harm and who engaged in suicide-related internet use also disclosed higher levels of suicidality compared with those who did not [8]. These findings mirror trends seen in general population studies, suggesting that suicide-related internet use is also linked to higher suicidal intent among mental health patients.

Despite these insights, research on suicide-related internet use in mental health patients remains scarce. To develop effective interventions, both web-based and offline, it is important to explore the characteristics of patients who use the internet for suicide-related purposes and examine whether they differ from those who do not. Examining which patients turn to suicide-related internet use while remaining in contact with mental health services can provide valuable information for clinical risk assessment and reveal potential gaps in knowledge on this type of behavior. In addition, this can help inform targeted web-based interventions as well as policies aimed at protecting at-risk people and promoting safer web-based environments. Therefore, this study aims to compare the sociodemographic, clinical, and suicidality characteristics of suicidal mental health patients who engage in suicide-related internet use with those who do not.

## Methods

### Study Design

This was a cross-sectional study using a web-based survey. The questionnaire was hosted on the Qualtrics platform from June 1, 2023, to December 31, 2023.

### Ethical Considerations

Participants provided informed consent to participate in the survey. All survey responses were anonymous and no compensation was provided for participants. Ethical approval was obtained from the University of Manchester Research Ethics Committee 1 on April 3, 2023 (ref: 2023-16133-28055).

### Eligibility Criteria

Inclusion criteria were presented at the start of the survey and consisted of (1) being aged 18 years and older, (2) being in contact with secondary mental health services in the past 12 months, (3) experiencing suicidal thoughts or behaviors in the past 12 months, (4) using the internet, (5) living in the United Kingdom, and (6) having a good command of the English language.

### Recruitment

We advertised the study on social media (X, Reddit, Facebook, BlueSky, Mastodon, TikTok, Instagram, and LinkedIn), University of Manchester staff and student mailing lists, newsletters of mental health and social care charities, and through webpages of mental health and wellbeing-related organizations, and radio. The recruitment materials were commonly shared throughout platforms. For participating in this study, the participants were not offered any financial incentives, such as monetary compensation or vouchers. Finally, participants were not asked to disclose any identifiable data (ie, name, email address, and geographical location), nor were their IP address data collected.

### Materials

The questionnaire was initially developed by the author [LB], based on existing literature and the author’s own experience of navigating online spaces. Coauthors [IH, SF, SI, and PT] and 2 members of a Patient and Public Involvement Group with lived experience of suicidality provided input on the phrasing of survey questions and design of the recruitment materials. There were 45 questions in total, most in multiple-choice format with free-text options to provide additional information. Participants were asked about lifetime psychiatric diagnoses and those received in the past 12 months due to the potentially high level of distress, self-stigmatization, and need for information following new diagnoses that might motivate patients to engage in suicide-related internet use [13,14]. For questions on the amount of support from family, friends, or community, satisfaction with mental health service provision, how intense their thoughts of suicide were, and the amount of planning involved in suicide attempt, participants provided answers using a visual analog scale (VAS). VAS scores ranged from 0 to 10 and were interpreted as follows: 0‐2, very low or minimal; 2.1‐4.0, moderate low; 4.1‐6.0, neutral or mixed effects; 6.1‐8.0, moderate high; and 8.1‐10.0, very high. There were 4 free-text questions relating to search terms to look for suicide-related information, disclosure of suicide-related internet use to clinicians, and any additional thoughts on suicide-related internet use participants might have had; qualitative findings from this free-text data will be presented elsewhere. To ensure data quality, the survey also included 1 attention-checking question. The full survey is available as part of the supplementary material (Multimedia Appendix 1).

### Procedure

**Figure 1.**
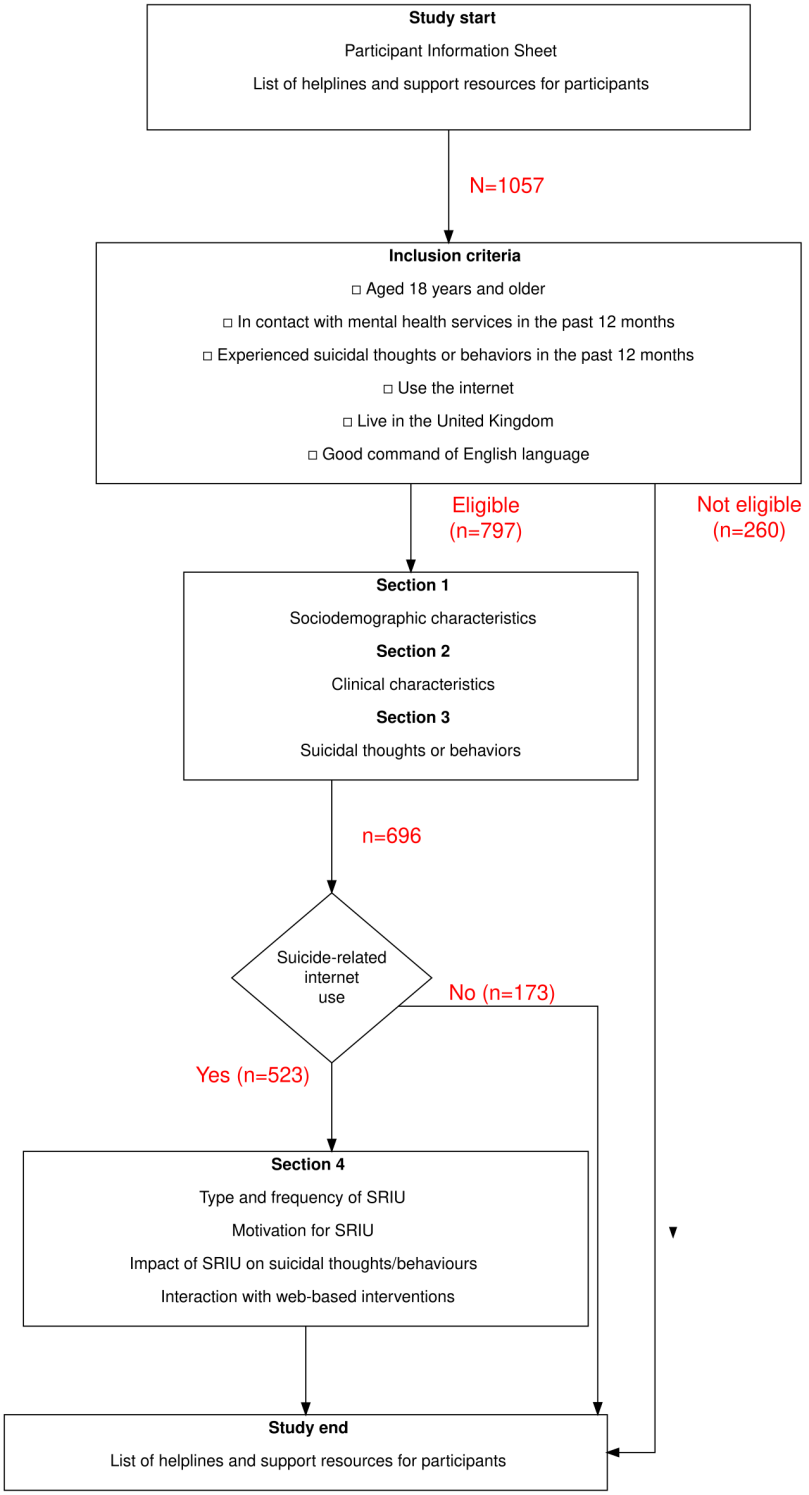
Study flowchart: inclusion criteria and sections of survey. SRIU: suicide-related internet use.

Before commencing the questionnaire, participants were presented with the study information sheet, and informed consent was obtained. A list of resources, such as helplines and services they could contact if they became distressed, was provided. The survey consisted of 4 sections (Figure 1). The first 2 sections covered sociodemographics and mental health history. The third section covered suicidal thoughts and attempts, adverse life events (ie, relationship breakdown and financial difficulties) that may have contributed to their suicidality, and perceived support received from friends, family, or their community. Trigger warnings for questions related to suicidal thoughts and attempts were provided. The fourth section covered suicide-related internet use defined as:

*Suicide-related internet use is any internet use related to your thoughts, feelings and behaviors connected to suicide. This kind of use can be, for example, expressing suicidal feelings or thoughts on social media or using the internet to get help and support when feeling suicidal.*

Participants were asked if they engaged in suicide-related internet use in the past 12 months. If they answered “no,” they were exited from the questionnaire. Those who answered “yes” continued with section 4 on suicide-related internet use. All participants were provided with a list of helplines and services.

### Participants

Out of a total of 1057 people who started the survey, 260 (25%) did not meet the inclusion criteria (72 did not answer inclusion criteria questions, 110 had no contact with secondary mental health services, 43 did not confirm suicidal thoughts or behaviors, and 35 failed to meet both inclusion criteria). Furthermore, 23 participants met the inclusion criteria but did not answer any questions. Of the remaining 797 participants, 23 were excluded based on missing data on all questions, 3 for failing the attention check question, and 5 for aberrant or joking answers. In total, 766 participants completed at least 3 sections of the survey (sociodemographic and clinical characteristics and suicidal thoughts or attempt, Figure 1), including 70 participants who did not provide information on whether they had used the internet for suicide-related purposes. As there was no way to interpolate this data, these 70 participants were excluded from the analysis. There were no differences in the sociodemographic and clinical characteristics and suicidal thoughts or attempts between those who provided information about their suicide-related internet use and those who did not. Therefore, there were 696 participants in the final analysis. Those who reported that they only came across suicide-related content accidentally (n=50) were grouped with those who reported they did not engage in suicide-related internet use, as those who accidentally came across suicide-related content did not actively seek it out, similar to those who did not engage in suicide-related internet use at all. There were no differences in the sociodemographic and clinical characteristics and suicidal thoughts or attempts between those who did not engage in suicide-related internet use and those who only came across suicide-related content accidentally.

### Statistical Analysis

Frequencies and percentages were used to present a descriptive analysis of categorical data. For data obtained through a visual analog scale, median and IQR were used for analysis as recommended [15]. The textual answers to open-ended questions were coded into existing variables or set up as new variables for quantitative analysis. The denominators in all estimates were the number of valid cases for the item, that is, if an item of information was not known for a case, that case was removed from the analysis of that variable. Comparisons between those who did and those who did not engage in suicide-related internet use were examined using the chi-square test for categorical variables and the Wilcoxon test for visual analog scales. Multivariable logistic regression with all variables that differed significantly between participants who did and did not engage in suicide-related internet use as predictors was carried out. We performed multiple imputations for multivariable logistic regression using chained equations; the direction and significance of the results, albeit with narrower CIs, were confirmed using this analysis (Multimedia Appendix 2). To compensate for multiple comparisons, Bonferroni correction was applied to all *P* values; therefore, *P* values less than .001 (.01/number of comparisons) were considered statistically significant. All analyses and designs of figures were carried out in RStudio (Posit PBC, version 2024.12.0.467) [16].

## Results

### Sociodemographic Characteristics

Of 696 participants, 523 (75.1%) had engaged in at least 1 type of suicide-related internet use in the past 12 months. Sociodemographic characteristics and comparisons of participants are presented in Table 1. Most were aged 25 years and over (553/696, 79.5%), with the most common age group being 25 to 34 years old (324/696, 46.4%) followed by 35 to 44 years (148/696, 21.3%). Almost two-thirds (444/696, 63.8%) were women, and 29.3% (204/696) were men. For 103/696 (14.8%) participants, gender did not match the sex assigned to them at birth. Most participants were of white ethnicity (611/696, 87.8%). Over half (384/696, 55.2%) of all participants were nonheterosexual. Most participants held higher education qualifications, with over half having a university degree (392/696, 56.3%). There was no statistically significant difference in sociodemographic characteristics between participants who did and did not engage in suicide-related internet use, even though almost twice as many participants aged 25 years or younger (23.1% vs 12.7%, *P*=.01) engaged in this behavior.

**Table 1.** Sociodemographic characteristics of mental health patients who did and did not engage in suicide-related internet use.

Characteristics	Engaged in SRIU (n=523)^a^, n (%)	Did not engage in SRIU (n=173), n (%)	Total (n=696), n (%)	Chi-square *(df)*	*P* value
Age group (years)					
18‐24	121 (23.1)	22 (12.7)	143 (20.5)	8.65 (2)	.01
25‐34	239 (45.7)	85 (49.1)	324 (46.6)	0.62 (2)	.74
35‐44	105 (20.1)	43 (24.9)	148 (21.3)	1.77 (2)	.41
45‐54	41 (7.8)	19 (11.0)	60 (8.6)	1.63 (2)	.44
55‐74	17 (3.3)	4 (2.3)	21 (3.0)	0.39 (2)	.82
Gender					
Female	337 (64.4)	107 (61.8)	444 (63.8)	0.38 (2)	.83
Male	149 (28.5)	55 (31.8)	204 (29.3)	0.68 (2)	.71
Other, prefer not to say	37 (7.1)	11 (6.4)	48 (6.9)	0.10 (2)	.95
Gender matching birth sex					
Yes	438 (83.7)	155 (89.6)	593 (85.2)	3.53 (2)	.17
No, nonbinary	45 (8.6)	9 (5.2)	54 (7.8)	2.10 (2)	.35
No, transgender	29 (5.5)	6 (3.5)	35 (5.0)	1.17 (2)	.56
Other, prefer not to say	11 (2.1)	3 (1.7)	14 (2.0)	0.09 (2)	.96
Ethnicity					
Asian, Asian British	17 (3.3)	3 (1.7)	20 (2.9)	1.07 (2)	.59
Black, African, Caribbean, Black British	4 (0.8)	4 (2.3)	8 (1.1)	2.74 (2)	.25
Mixed, multiple ethnic group	30 (5.7)	11 (6.4)	41 (5.9)	0.09 (2)	.96
White, White British, White Irish	459 (87.8)	152 (87.9)	611 (87.8)	<0.01 (2)	.99
Other, prefer not to say^b^	13 (2.5)	3 (1.7)	16 (2.3)	0.33 (2)	.85
Sexual orientation					
LGB	196 (37.5)	60 (34.7)	256 (36.8)	0.44 (2)	.80
Heterosexual	219 (41.9)	93 (53.8)	312 (44.8)	7.42 (2)	.02
Other, prefer not to say	108 (20.7)	20 (11.6)	128 (18.4)	7.16 (2)	.03
Highest level of education					
A or AS levels or highers or advanced highers and less	165 (31.5)	46 (26.6)	211 (30.3)	1.51 (2)	.47
University degree and above	291 (55.6)	101 (58.4)	392 (56.3)	0.40 (2)	.80
Other, prefer not to say^c^	67 (12.8)	26 (15.0)	93 (13.4)	0.55 (2)	.76
Employment status					
In paid employment (including part-time, self-employed)	264 (50.5)	103 (59.5)	367 (52.7)	4.28 (2)	.12
Unemployed	100 (19.1)	20 (11.6)	120 (17.2)	5.21 (2)	.07
Full-time student	48 (9.2)	19 (11.0)	67 (9.6)	0.49 (2)	.78
On long-term sick leave	77 (14.7)	18 (10.4)	95 (13.6)	2.06 (2)	.36
Other, prefer not to say	34 (6.5)	13 (7.5)	47 (6.8)	0.21 (2)	.90
Relationship status					
Single	256 (48.9)	67 (38.7)	323 (46.4)	0.06 (2)	.97
In a relationship (not cohabiting)	60 (11.5)	21 (12.1)	81 (11.6)	5.46 (2)	.07
Married, cohabiting, civil partnership	166 (31.7)	77 (44.5)	243 (34.9)	9.33 (2)	.009
Separated, divorced	23 (4.4)	6 (3.5)	29 (4.2)	0.28 (2)	.87
Other, prefer not to say	18 (3.4)	2 (1.2)	20 (2.9)	2.43 (2)	.30

a SRIU: suicide-related internet use.

b includes asexual, pansexual, and questioning.

c includes homemaker, training scheme, and retired.

### Clinical and Mental Health Service Characteristics

Those who engaged in suicide-related internet use were more likely to receive a diagnosis of personality disorder compared with those who did not (34.4% vs 18.5%, *P*<.001; Table 2). Most participants had been prescribed psychotropic medication in the last 12 months (554/696, 79.6%), and 112/696 (16.1%) had been admitted for psychiatric in-patient care. When asked about their satisfaction with their mental health service provision, participants were, on average, moderately dissatisfied (VAS score median=4.0, IQR=2.0‐6.3); this did not differ between those who did and did not engage in SRIU nor between different diagnoses.

**Table 2.** Mental health diagnoses of patients who did and did not engage in suicide-related internet use.

	Engaged in SRIU (n=523)^a^	Did not engage in SRIU (n=173)	Total (n=696)	Chi-square *(df)*	*P* value
No diagnosis	108 (20.7)	43 (24.9)	151 (21.7)	1.30 (2)	.52
Schizophrenia and other primary psychotic disorders	41 (7.8)	12 (6.9)	53 (7.6)	0.18 (2)	.92
Affective disorder^b^	378 (72.3)	111 (64.2)	489 (70.3)	5.17 (2)	.08
Anxiety, Phobia, OCD^c^	297 (56.8)	102 (59.0)	399 (57.3)	0.14 (2)	.93
PTSD^d^	191 (36.5)	59 (34.1)	250 (35.9)	0.44 (2)	.80
Eating disorder	117 (22.4)	29 (16.8)	146 (21.0)	2.66 (2)	.27
Alcohol or drug dependence	87 (16.6)	19 (11.0)	106 (15.2)	3.39 (2)	.18
Personality disorder	180 (34.4)	32 (18.5)	212 (30.5)	16.28 (2)	<.001^e^
Autism spectrum disorder	137 (26.2)	32 (18.5)	169 (24.3)	4.47 (2)	.11
ADHD^f^	91 (17.4)	40 (23.1)	131 (18.8)	2.62 (2)	.27
Other	87 (16.6)	26 (15.0)	113 (16.2)	0.30 (2)	.86
First diagnosis received in the last 12 months	69 (13.2)	31 (17.9)	100 (14.4)	2.24 (2)	.33

a SRIU: suicide-related internet use.

b depressive illness and bipolar disorder.

c OCD: obsessive-compulsive disorder.

d PTSD: posttraumatic stress disorder.

e *P*≤.001.

f ADHD: attention deficit hyperactivity disorder.

### Suicidal Thoughts and Behaviors

One of the inclusion criteria was having experienced suicidal thoughts in the past 12 months; therefore, all participants had contemplated suicide. However, 19 participants (19/696, 2.7%) chose not to provide additional information about their suicidal thoughts. Twelve months before completing the survey, 507/677 (74.9%) participants had thoughts of suicide at least once a week, including 214/677 (31.6%) who had thoughts of suicide daily or almost daily. Participants who had engaged in suicide-related internet use were more likely to have thoughts of suicide every day or almost every day compared with those who did not engage in this behavior (35.0% vs 17.9%, *P*<.001). The average intensity of thoughts of suicide for all participants was moderately high (median=6.4, IQR=4.9‐7.6); those who had engaged in suicide-related internet use were more likely to report higher intensity of suicide thoughts compared with those who did not engage in it (VAS score median=6.6, IQR=5.0‐7.8, vs VAS score median=5.1, IQR=4.0‐7.0, *P*<.001). Fifteen percent of participants (91/585, 15.5%) had not disclosed their thoughts of suicide to anyone; those who had engaged in suicide-related internet use were significantly more likely to disclose this compared to those who had not engaged in this behavior (87.8% vs 72.8%, *P*<.001). The majority of those who did (460/585, 79%) disclosed their thoughts of suicide to a mental health professional.

A quarter of participants reported attempting suicide in the last 12 months (186/696, 26.7%); this was more prevalent among those who had engaged in suicide-related internet use than those who did not (32.5% vs 9.2%, *P*<.001). Of those who had attempted suicide, over half (105/186, 56.5%) had done so more than once and 113/186 (60.8%) had subsequently been hospitalized. Participants who attempted suicide had, on average, reported moderately high levels of planning involved in the attempt (VAS score median=6.7, IQR=4.0‐8.25). Most of those who attempted suicide did not disclose their plans to anyone (112/186, 60.2%).

Life events that were reported as contributing to suicidality were most commonly relationship difficulties with a partner or family (230/626, 36.7%), serious financial difficulties (193/626, 30.8%), and insomnia (180/626, 30.2%). There were no significant differences in reported life events between those who did and did not engage in suicide-related internet use, even though it seems that more patients who engaged in suicide-related internet use experienced insomnia (32.9% vs 21.9%, *P*=.01) and violence as victims (24.4% vs 13.5%, *P*=.01). When asked about the support for their mental health they had received from their friends, family, and community, participants overall reported a mixed level of support (VAS score median=5.8, IQR=3.1‐8.0); there was no significant difference in perceived levels of support between those who did and did not engage in suicide-related internet use.

### Logistic Regression

All significant variables from the descriptive comparisons were entered together in a multivariable logistic regression model with engagement in suicide-related internet use as the outcome. Patients who engaged in suicide-related internet use were twice as likely to have received a diagnosis of personality disorder and to disclose their thoughts of suicide to someone, and almost 3 times as likely to attempt suicide compared with patients who did not engage in this behavior (Table 3).

**Table 3.** Logistic regression model of predictors of engaging in suicide-related internet use among mental health patients (n=652).

	Adjusted ORs^a^ (95% CI^b^)	*P* value
Diagnosis of personality disorder	1.94 (1.22‐3.08)	<.004
Thoughts of suicide every day	1.63 (1.03‐2.58)	.03
Disclosed thoughts of suicide to someone	1.99 (1.22‐3.25)	.007
Attempted suicide within the last 12 months^c^	2.81 (1.55‐5.11)	<.001
Intensity of suicidal thoughts	1.11 (1.01‐1.23)	.03

a OR: odds ratio.

b Confidence intervals.

c continuous variable.

## Discussion

### Principal Findings

In our UK sample, three-quarters of participants with suicidal thoughts or behaviors who were aged 18 years or older and in contact with mental health services in the past 12 months had engaged in suicide-related internet use. This suggests that suicide-related internet use is a common behavior among suicidal mental health patients who use the internet. There were no differences in sociodemographic factors between those who did and those who did not engage in suicide-related internet use; this provides additional evidence that suicide-related internet use is not a behavior present in young people only [10], underlying the need for clinicians to be aware of the potential for patients of all ages to engage in this behavior. Further findings indicate an association between suicide-related internet use and suicide attempts, personality disorder diagnosis, and disclosure of suicidal thoughts. Multivariable logistic regression revealed that suicide-related internet use was associated with a diagnosis of personality disorder, disclosure of suicidal thoughts, and suicide attempts in the past year.

Our observed proportion of suicide-related internet use is notably higher than what has been reported in similar studies [2-5]. There are several potential reasons for this finding. One possibility is that the usage of the internet increased over time, further boosted by the widespread adoption of smartphones and an increase in the time spent on the web since the COVID-19 pandemic [17,18]. This has made internet access more prevalent and immediate, particularly when compared with studies conducted nearly a decade ago. Alternatively, and perhaps more critically, our sample is unique in that it comprises people who had contact with mental health services. Unlike earlier studies that generally did not include mental health patients, our participants may have been more inclined to engage in suicide-related internet use as a means of supplementing their ongoing mental health care or during periods of distress. This difference could be a key factor contributing to the higher observed prevalence of suicide-related internet use in our study.

Although sociodemographic characteristics did not differ between those who did and those who did not engage in suicide-related internet use, our findings provide important insights into the characteristics of patients who engaged in this behavior. The majority of those who have engaged in suicide-related internet use were women, likely due to their higher survey participation rates [19,20] and the gender paradox in suicide, where women experience more suicidal ideation despite lower suicide mortality rates. Nonetheless, the higher prevalence of women in our sample could also be due to women engaging more frequently in suicide-related internet use. Similarly, our sample was also highly educated, with over half holding a university degree. While those who engage in suicide-related internet use may be more educated, survey participation is generally higher among highly educated people [21]. Despite 85% of all participants aged 25 years or younger engaging in suicide-related internet use, the overall age distribution demonstrates the presence of this behavior in all age groups. This supports the notion that suicide-related internet use is not limited to younger age groups [10]. In addition, a notable proportion of participants identified as gender or sexual minorities. This may be because LGBTQ people have higher rates of suicidality compared with their heterosexual and cisgender peers [22]. LGBTQ people are often more reliant on web-based platforms for mental health support due to systemic barriers in traditional care settings. These barriers include pervasive experiences of discrimination, microaggressions, and a lack of culturally competent care within mental health systems [23,24]. Consequently, many turn to the internet not only for information but also to find community, validation, and anonymity, which can be particularly important in navigating sensitive topics such as suicidality. Specifically, transgender and nonbinary people often encounter additional barriers such as being misgendered, denied care, or subjected to pathologization in clinical settings, which can further drive them to seek alternative support online [25]. In addition, participation in surveys may be viewed by some LGBTQ people as a form of advocacy or community contribution: an opportunity to shed light on mental health disparities affecting their communities [26]. Most participants in the study identified as white, with a notable minority of those with a mixed heritage background; this mirrors the ethnic composition of suicide rates in both general and patient populations [27,28]. However, suicidality among ethnic minority groups in the United Kingdom remains under-researched [28], and this gap seems to also extend to suicide-related internet use.

Clinically, most participants were diagnosed with affective or anxiety disorders, similar to the general patient population [29]. Among those who attempted suicide, over half were subsequently hospitalized, highlighting the severity of suicidal intent in our sample. In addition, our participants were moderately dissatisfied with the mental health service provision they received, which may have contributed both to the higher prevalence of suicide-related internet use and participation in our survey, as those dissatisfied with care provision might have been more inclined to engage in suicide-related internet use and to participate in the study. A similar situation was true for support for their mental health they had received from their friends, family, and community; mixed levels of perceived support may have contributed to the higher prevalence of suicide-related internet use in our sample. Notably, even though not significant due to a more stringent significance cut-off value, almost a third of patients who have engaged in suicide-related internet use experienced insomnia, and a further quarter experienced violence as a victim: these characteristics warrant further investigation.

In general, our sample was highly suicidal, with almost three-quarters of participants experiencing thoughts of suicide every day, and we found a greater likelihood of suicide attempts in the past year and an increased intensity of suicidal thoughts among mental health patients who engaged in suicide-related internet use. This supports findings from studies conducted on both general [2-6] and clinical populations [12,30]. A high intensity of suicidal thoughts can point to a higher risk of suicide attempts among patients who have engaged in suicide-related internet use, but it can also show how increasing suicidality can lead people to seek new web-based help resources and ways to cope [2,4,6]. Indeed, findings from another piece of research informed by this dataset show that for participants in our sample, the most common motivations for engaging in suicide-related internet use were searching for help and searching for suicide methods [31]. Therefore, the higher prevalence of engaging in suicide-related internet use in those with more frequent and intense thoughts of suicide may indicate help and support-seeking intentions. On the other hand, there is a possibility that suicide-related internet use engagement may have informed participants’ suicide attempts; previous research has shown that suicide attempts of mental health patients were often shaped by their web-based searches [10,30], with evidence showing that these searches were directly connected to suicide attempts in 58%‐65% of patients [32,33]. This underscores the potential role of the internet in planning and facilitating suicide attempts, a significant concern given the influence web-based searches can make in the selection of novel and highly fatal suicide methods [10]; however, this was not examined in this study.

Those who engaged in suicide-related internet use were more likely to have received a diagnosis of personality disorder compared with those who did not engage in suicide-related internet use. While, to the authors’ knowledge, there is currently no research on personality disorders and suicide-related internet use, research on personality disorders and internet use, in general, has shown a higher incidence of internet addiction and problematic social media use, such as using social media as a distraction from interpersonal problems, reassurance seeking, and self-confidence issues in people meeting criteria for personality disorder [34,35]. This may indicate that people with personality disorders are more vulnerable to developing maladaptive web-based behaviors, including suicide-related internet use. Given that personality disorders are often characterized by difficulties in emotional regulation, impulsivity, and unstable self-image [36], it is plausible that these people may turn to suicide-related internet use as a coping mechanism, to manage distress, or to seek validation. In addition, while no differences in satisfaction with services were found between diagnostic groups in our sample, overall satisfaction with service provision was low. Research has shown that patients with personality disorders often experience challenges in mental health service provision [37] due to the commonly present stigma associated with these diagnoses and the tendency for it to obscure the individual needs of the patient [38,39]. As a result, mental health patients diagnosed with personality disorders may turn to the internet for suicide-related purposes when they do not feel adequately supported by services. Future research should explore the specific mechanisms underlying the relationship between personality disorder diagnosis and suicide-related internet use to better understand its potential risks and implications.

Mental health patients who used the internet for suicide-related purposes were more likely to disclose their thoughts of suicide to someone compared to those who did not, most often a mental health professional. A recent meta-analysis showed that less than half (44%) of mental health patients who have experienced suicidal thoughts or behaviors disclosed this to someone [40]; the prevalence of disclosure of suicidal thoughts was much higher in our sample (84%). One reason for this might be the self-selection of our participants, that is, those who were more likely to disclose their suicidal thoughts and behaviors by participating might also be more likely to disclose this to others. However, 2 studies reported that those who were motivated by seeking help to engage in suicide-related internet use were less likely to disclose their issues with suicide or self-harm and to seek treatment offline [2,41]. As both of these studies concerned this behavior in young people, our results offer potential evidence that the interaction between suicide-related internet use and disclosure may be different in adults.

### Strengths and Limitations

To the authors’ knowledge, this is one of the first national studies to focus on suicide-related internet use in suicidal adults in recent contact with mental health services. While previous research has predominantly examined this behavior in younger populations, this study brings more insight into the suicide-related internet use of adults. A further strength of this study lies in the comparatively large sample of participants; notably, our sample size is 3 to 5 times larger than in previous studies, potentially enhancing the representativeness of our findings.

However, the results should be considered in light of certain limitations, most of which are common for cross-sectional survey studies in general. First, the cross-sectional design means we cannot establish causality. Second, our study sample consists of UK-based people in contact with secondary mental health services. Therefore, the generalizability of our findings to other nations is limited by differences in internet infrastructure, suicide prevention resources, and access to mental health care. The results may also not be generalizable to primary care patients and people without diagnoses of mental illness. Third, the self-selection bias of our participants, manifesting in over- and under-representation of certain groups, potentially impacted several aspects of the results. Therefore, we are unable to generalize our results to all suicidal mental health patients. Specifically, the comparisons between those who did and those who did not engage in suicide-related internet use were impacted by unequal group sizes (3:1 ratio), which was partially addressed using Bonferroni correction. Overall, results suggest that suicide-related internet use is a common behavior among suicidal mental health patients who use the internet, though it is important to interpret the findings with caution as this prevalence is based on participants’ self-report. The study’s retrospective design could have introduced recall bias. However, we attempted to mitigate this by focusing on events within a consistent and short time frame (the past 12 mo). While the lack of standardized measures in our study limits comparability, we believe that it brings additional strengths by reducing recall bias and socially desirable responding. However, we could not verify participants’ reported mental health histories against official records.

Another limitation is the lack of measurement regarding the temporal relationship between suicidal thoughts or attempts and suicide-related internet use, namely, whether suicide-related internet use preceded, coincided with, or succeeded suicidal thoughts or attempts. For instance, suicide-related internet use may precede a suicidal attempt. This temporal pattern would suggest a critical window for early intervention, where identifying and responding to such web-based behaviors could help mitigate the progression toward more severe mental health crises. Early detection through digital trace data could enable mental health professionals, platforms, or support systems to offer timely resources or outreach before suicidal ideation fully develops. Alternatively, if suicide-related internet use follows a suicide attempt, it may reflect a person’s need to seek understanding, share their experiences, or make sense of what happened. This could also indicate postattempt distress, confusion, or a continued risk of harm. However, suicide-related internet use is often not a single event; rather, it tends to occur repeatedly over time as people may engage in multiple instances and types of this behavior [30]. Furthermore, suicidal people may engage in suicide-related internet use during varying stages of distress, sometimes as a means of coping or information-seeking and at other times as a response to immediate crises. Consequently, this complexity makes it challenging to determine which behaviors precede or succeed others, suggesting the need for alternative study designs, such as longitudinal or ecological momentary assessment methods, to capture the temporal aspect of this behavior.

A recent scoping review called for the increased use of validated questionnaires in the field of suicide-related internet use research [42]. While we agree that using validated questionnaires increases reliability and comparability across studies, there is currently no validated tool that comprehensively addresses suicide-related internet use, especially not for our target population of adult mental health patients [42]. Consequently, we maintain that the current questionnaire, developed with input from Patient and Public Involvement and Engagement, meets rigorous standards of scrutiny for this study.

### Conclusion

By recognizing suicide-related internet use as a prevalent behavior among high-risk groups, mental health professionals can be better equipped to identify it and engage with patients. As recommended in previous work by this group [10,43], a direct, nonjudgmental inquiry on whether and why a patient has engaged in suicide-related internet use should be a part of the assessment in mental health services. Our findings on high levels of suicidal ideation disclosure, particularly to clinicians, suggest a possible pathway for suicide-related internet use inquiry; once a patient discloses suicidal thoughts to a mental health professional, a follow-up inquiry about suicide-related internet use could be beneficial. An open discussion about suicide-related internet use and the role it fulfills in a patient’s life, whether as an additional coping mechanism or a supplement to service provision, may help clinicians discover any unmet needs. Through a collaborative approach, clinicians and patients can explore effective ways to address these needs. This approach has the potential to enhance therapeutic outcomes, reduce suicide risk, and improve the quality of mental health care for vulnerable people.

Our previous findings underscore the importance of ensuring that people engaging in suicide-related internet use encounter supportive, educational, and appropriately tailored web-based content [31]. Given the widespread nature of such searches, suicide prevention results represent a critical first line of defense. However, their effectiveness may depend on algorithmic sensitivity, as well as the integration of multimodal crisis services and educational materials. Furthermore, our previous study revealed that many users lack awareness of how to navigate suicide-related content safely, highlighting the need to improve digital literacy. This aligns with previous work identifying mental health literacy gaps as barriers to effective web-based help-seeking and suggests a dual approach: integrating adaptive web-based interventions while empowering clinicians to address suicide-related internet use directly and constructively in care settings [31].

## Supplementary material

10.2196/73702 Multimedia Appendix 1 Questionnaire.

10.2196/73702 Multimedia Appendix 2 Multiple imputation - sensitivity analysis

## Abbreviations

VAS: visual analog scale
