# Supplementary material for: Characteristics of Suicidal Patients Who Engaged in Suicide-Related Internet Use in the United Kingdom: Cross-Sectional Survey Findings
Source: JMIR Ment Health. 2025 Sep 5;12:e73702. doi: 10.2196/73702 (PMC12413185; doi:10.2196/73702)
Supplement: Multimedia Appendix 2 [file mental-v12-e73702-s002.docx]

Appendix 2. Multiple imputation- sensitivity analysis

Multiple imputation using chained equations (MICE) was conducted on the dataset. MICE uses a different conditional distribution for the imputation of each variable. Ten multiply imputed datasets have been created and the results were pooled using Rubin's rules to produce pooled estimates. The analysis was performed using RStudio, version 2024.12.0.467. Results are presented in Table 1.

Table 1. Logistic regression model of predictors of engaging in suicide-related internet use among mental health patients (N=696): imputed dataset

|  | Adjusted ORs (95%CI) | p-value |
| --- | --- | --- |
| Diagnosis of personality disorder | 1.74 (1.12 - 2.71) | 0.012 |
| Thoughts of suicide every day of almost every day | 1.66 (1.06 - 2.6) | 0.024 |
| Disclosed thoughts of suicide to someone | 2.1 (1.31 - 3.36) | 0.002 |
| Attempted suicide within the last 12 months^a^ | 2.83 (1.59 - 5.04) | < 0.001 |
| Intensity of suicidal thoughts | 1.14 (1.04 - 1.25) | 0.006 |
| ^a^continuous variable | | |
